# Cost-effectiveness of nirsevimab and maternal RSVpreF for preventing respiratory syncytial virus disease in infants across Canada

**DOI:** 10.1186/s12916-025-03928-z

**Published:** 2025-02-21

**Authors:** Samara Bugden, Shweta Mital, Hai V. Nguyen

**Affiliations:** 1 School of Pharmacy, Memorial University of Newfoundland, 300 Prince Philip Drive, St. John’s, NL A1B 3V6 Canada; 2 College of Pharmacy, Apotex Centre, University of Manitoba, 750 McDermot Avenue West, Winnipeg, MB R3E 0T5 Canada

**Keywords:** Respiratory syncytial virus, Cost-effectiveness analysis, Monoclonal antibodies, Maternal vaccination

## Abstract

**Background:**

Nirsevimab, a long-acting monoclonal antibody, and RSVpreF, a maternal vaccine, are newly approved respiratory syncytial virus (RSV) prophylactics for infants in Canada. Both have the potential to expand prevention efforts, but there is limited evidence regarding their cost-effectiveness and how it varies across the country, despite disparate hospitalisation rates and resource use among different populations.

**Methods:**

We developed a decision tree model to follow twelve monthly birth cohorts through their first year of life, incorporating risk differentiation based on Canadian region, prematurity, and comorbidities. The model tracked medically attended infections, including hospitalisations, intensive care unit admissions, and outpatient visits, comparing costs (in 2024 Canadian dollars) and effectiveness (in quality-adjusted life years (QALYs)) of nine different immunisation strategies compared to no intervention. The analysis was conducted from both healthcare and societal perspectives. We conducted threshold price analyses, varying the price-per-dose of each product to determine the threshold prices at which expanded coverage becomes cost-effective.

**Results:**

At base case prices, the optimal strategy varies by region, but in all cases, the optimal strategy is both cost-saving and more effective than no intervention. In southern Canada, it is optimal to immunise only palivizumab-eligible infants (those born very prematurely or with high-risk comorbidities) with nirsevimab, resulting in cost savings of $4.14 and QALY gains of 0.000022 QALY per infant compared to no intervention. In the Northwest Territories, it is best to expand protection with nirsevimab to include all preterm infants (cost savings of $28.68 and QALY gains of 0.00007 per infant). In Nunavik and Nunavut, immunising all infants under 6 months and all infants under twelve months with nirsevimab are the best strategies, respectively (cost savings of $399.61 and QALY gains of 0.000821 per infant in Nunavik, and cost savings of $1067.03 and QALY gains of 0.000884 per infant in Nunavut). Universal, country-wide immunisation with nirsevimab would require a price-per-dose of under $112 to become the most cost-effective prevention strategy.

**Conclusions:**

The optimal strategy for preventing respiratory syncytial virus disease in Canadian infants depends on product price and regional risk level and resource use. Canadian policy should account for these factors.

**Supplementary Information:**

The online version contains supplementary material available at 10.1186/s12916-025-03928-z.

## Background

Respiratory syncytial virus (RSV) infects virtually all children before the age of two [1]. Most infections are mild and self-limiting, but some progress to lower respiratory tract infections (LRTI) such as bronchiolitis or pneumonia and require hospitalisation. Globally, RSV is the primary cause of LRTIs in children under five, with the majority of hospitalisations occurring in infants under 1 year [2].

Palivizumab, a monoclonal antibody, has been the only available defense against RSV disease in infants for the last twenty years. It must be given monthly, requiring five doses to extend protection through a full RSV season [3]. Its price and frequency of administration mean it can cost up to $10,000 to protect a single infant against RSV for one season in Canada [4]. Consequently, palivizumab is only given to high-risk infants, i.e., young, premature infants and infants with comorbidities such as congenital heart disease (CHD) and chronic lung disease of prematurity (CLD) [3]. This leaves the majority of infants unprotected. Many prophylactic candidates for RSV are in development to fill this gap.

The United States Food and Drug Administration (FDA) and Health Canada have recently approved two prophylactics for preventing RSV disease in infants. Nirsevimab is a long-acting monoclonal antibody similar to palivizumab, with the distinct improvement that a single dose offers protection for an entire RSV season [5, 6]. The RSVpreF vaccine can be given to pregnant women in their third trimester, conferring immunity through transplacental antibody transfer [7].

While pricing and coverage decisions are pending in Canada, in the USA both nirsevimab and RSVpreF are considerably cheaper per dose than even a single dose of palivizumab [8]. The lower price and comparative ease of single-dose administration for these products offer the potential to expand prevention efforts beyond the highest-risk infants. Both the United States’ Centers for Disease Control and Prevention (CDC) and the National Advisory Committee on Immunization (NACI) in Canada have recommended broad protection [9, 10], but the cost-effectiveness of this expansion is still unclear. Nirsevimab has a higher efficacy than RSVpreF, but RSVpreF is cheaper under current US prices. RSVpreF requires 2 weeks for maternal antibody transfer [7]; infants born before this time may not be fully protected and this could necessitate backup coverage with nirsevimab after birth. Questions remain as to which infants (if not all) should receive prophylaxis, and which prophylactic should be administered.

Infants in Canada’s remote northern Inuit communities have documented RSV hospitalisation rates of two to 17 times that of southern Canada [11, 12]. The reason for this disparity is uncertain, but postulated factors include overcrowding, lower breastfeeding rates, higher smoking rates, and the many other inequalities in social determinants of health faced by indigenous communities [11, 13]. A genetic predisposition to severe RSV disease in Inuit infants has also been posited [14]. Compounding these higher hospitalisation rates is the fact that hospitalising infants from remote northern communities can involve expensive medical evacuations.

Canadian guidelines for palivizumab eligibility include specifications for comorbidities, prematurity, and regional risk levels [3]. Guidelines for new prophylactics should similarly consider these factors, but no existing study has fully incorporated them into their cost-effectiveness analyses. A study by Shoukat et al. [15] was based on Ontario demographics only, with no consideration of northern communities, while Gebretekle et al. [16] differentiated infants only by prematurity, and not by comorbidity risk level. Gebretekle et al. ran a scenario analysis to broadly address the entire Canadian north, using hospitalisation rates based on Alaskan data, but hospitalisation rates and transportation costs vary widely between northern regions and an analysis with more detailed regional differentiation is needed. In addition, US guidelines for RSVpreF recommend seasonal administration [9]; neither study included any strategies involving seasonal RSVpreF administration in their analyses.

Due to these gaps, the optimal strategy for RSV prevention in infants across Canada is still unknown. We aimed to address this by conducting a cost-effectiveness analysis of nirsevimab and RSVpreF through a range of strategies, with differentiation for prematurity, comorbidities, and regional risk, including southern Canada and three northern Canadian regions: the Northwest Territories, Nunavut, and Nunavik, Quebec.

## Methods

### Target population

The target population of this study was Canadian infants under 1 year of age. Infants under 6 months, infants born prematurely, and infants with CHD or CLD have an increased risk of severe RSV disease [3], as do infants born during the RSV season. While the disease burden of RSV remains high in older children, particularly in outpatient settings [17], there are currently no prophylactic options for children beyond infancy.

The population was first divided into monthly birth cohorts to capture seasonal risk variation. The RSV season was assumed to span from October to March in southern Canada and from December to May in northern Canada [18]. The population was then subdivided into risk groups based on the level of prematurity (33–36 weeks of gestational age, < 33 weeks of gestational age), and the presence of CHD or CLD. We considered low-risk infants to be those born at ≥ 37 weeks of gestational age (wGA) without comorbidities.

### Model Structure

**Fig. 1:**
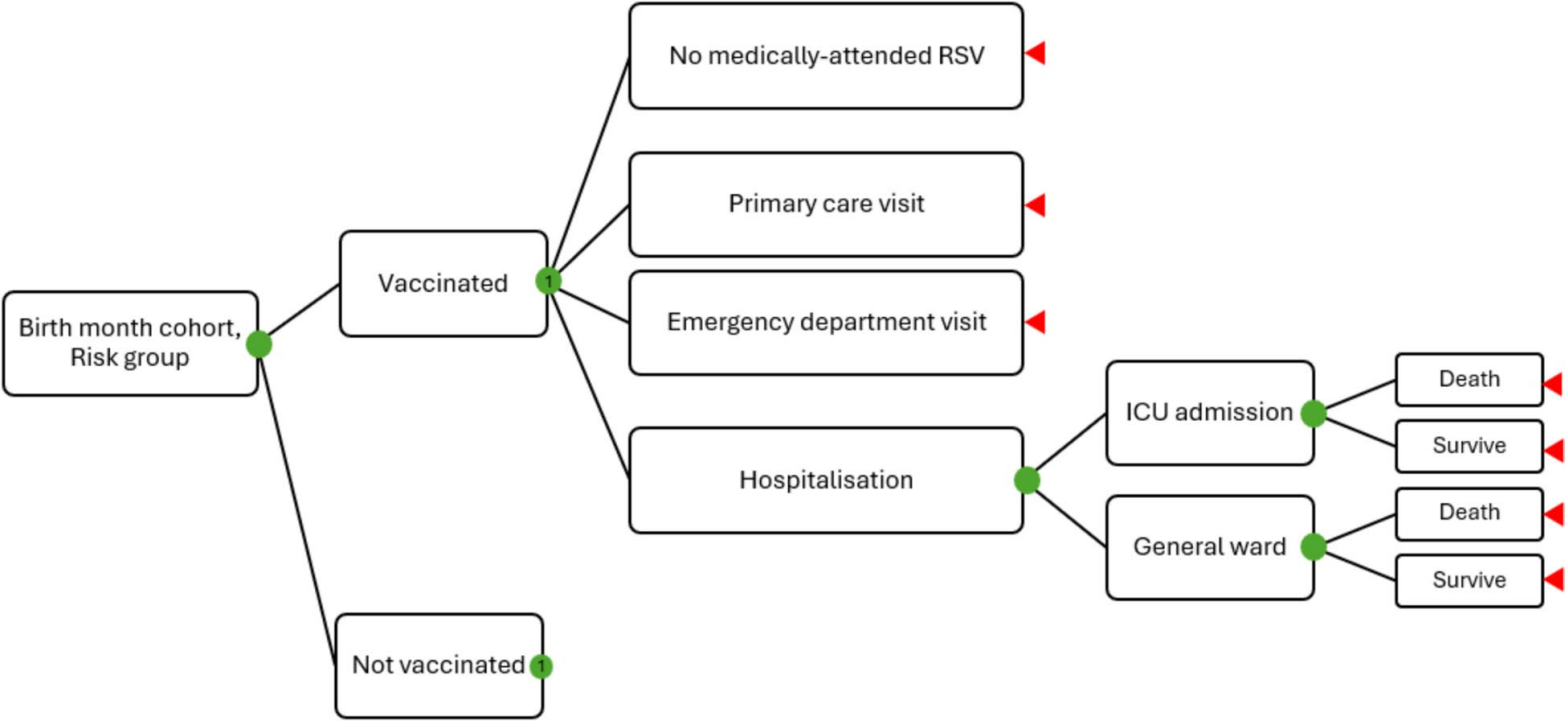
Decision tree. Branches from the vaccinated node (1) are cloned at the not vaccinated node. Pathways are replicated for each birth month cohort, prematurity/comorbidity risk group, Canadian region, and prevention strategy

We developed a decision tree using TreeAge Pro 2024 R 1.1 (Fig. 1) to follow twelve monthly birth cohorts through their first year of life, with risk differentiation for the Canadian region, prematurity, and comorbidities. The model tracked medically-attended infections including hospitalisations, intensive care unit admissions, and outpatient visits. The time horizon of the analysis was 1 year. The efficacy of nirsevimab is not established past 150 days [6], and the efficacy of RSVpreF is not established past 360 days [7]. Both prophylactics constitute passive immunisation, since infants receive maternal antibodies after their mothers are actively immunised with RSVpreF, rather than establishing a direct immune response themselves, and neither product is expected to confer long-term protection.

### Immunisation strategies

Nine strategies were evaluated. To approximate the current standard of care in Canada, we considered a strategy of giving palivizumab (PVZ) to “high risk” infants: those with CLD or CHD, and those born at < 33 wGA and less than 6 months old at the start of their first RSV season (strategy 1). For nirsevimab, we first considered a direct replacement of palivizumab by administering nirsevimab to high-risk infants only (NIRS HR) (strategy 2). We then considered expanding coverage to include infants at progressively lower risk of severe RSV infection, reflecting the fact that infants over 6 months old have much lower rates of hospitalisation than infants under 6 months old [12]. We first considered expanding coverage to infants born at 33–36 wGA and less than 6 months at season start (NIRS HR + MR) (strategy 3), then all infants less than 6 months at season start (NIRS < 6) (strategy 4), and finally the entire birth cohort (NIRS ALL) (strategy 5). For RSVpreF, we considered two strategies: seasonal administration (i.e. immunising women with in-season due dates) (ABR SEASONAL) (strategy 6) and year-round administration (ABR ALL) (strategy 7). Since 2 weeks are needed for transplacental antibody transfer [7], we also considered two combination strategies where RSVpreF administration was augmented with nirsevimab given to all preterm infants who may not have received the full benefit of RSVpreF, and to infants with CHD or CLD who are at higher risk of severe RSV infection. First, we augmented seasonal RSVpreF with nirsevimab (ABR SEASONAL + NIRS) (strategy 8), and then we augmented year-round RSVpreF with nirsevimab (ABR ALL + NIRS) (strategy 9).

### Model inputs

Incidence rates and probabilities for primary care visits, emergency department visits, hospitalisations, and ICU admissions are available with monthly granularity from birth to 11 months [12, 19–21]. RSV rates peak during the winter months in Canada; the seasonality of RSV was incorporated using a monthly distribution of hospitalisations [12]. Baseline hospitalisation rates were multiplied by incidence rate ratios to account for the higher risk of hospitalisation for premature babies and babies with CHD or CLD.

For the northern regions, the model incorporated both the higher incidence rates of hospitalisation for infants under 1 year old (15.8, 60.2, and 58.1 per 1000 live births for the Northwest Territories, Nunavut, and Nunavik, respectively) [11], and the higher transportation costs incurred with hospitalisations, based on the cost of a medical air evacuation to a regional or tertiary hospital and an economy return flight. Outpatient visit rates and hospitalisation outcomes were kept the same as southern Canada. We incorporated higher rates of preterm births seen in Nunavut and Nunavik, with CLD rates increasing as a consequence of more prematurity. For CHD, we assumed equal rates between regions in the absence of current, region-specific data. Hospitalisation rates and costs were not available for the Yukon territory, so our analysis omitted this region.

In the absence of Canadian drug costing information, costs for nirsevimab and RSVpreF were set to the United States’ CDC vaccine contract prices [8] for the base case (after conversion to Canadian dollars). For each prophylactic, we used three measures of efficacy: efficacy against medically attended (MA) RSV infection, efficacy against hospitalisation, and efficacy against ICU admission. There is no evidence to suggest differing efficacy between low- and high-risk infants for nirsevimab or RSVpreF. In the case of combination strategies where some infants received both products, the higher efficacy between the two products was applied. Full details on the sourcing and calculation of model inputs can be found in Additional File 1, Supplementary Material: Methods [3, 7, 8, 11, 12, 15, 19–50].

### Cost-effectiveness analysis

All costs were measured in 2024 Canadian dollars, and effectiveness was measured in quality-adjusted life years (QALYs). No discounting was necessary due to the 1-year time horizon. For each region, we calculated the incremental cost-effectiveness ratio (ICER) of every strategy, assessed as the incremental cost divided by the incremental effectiveness relative to another strategy. Strategies with an ICER less than a willingness-to-pay (WTP) threshold of $100,000 per QALY were considered cost-effective.

We conducted one-way sensitivity analyses to determine the impact of parameter uncertainty on the results. Since there is more uncertainty regarding hospitalisation rates in the northern regions than in southern Canada, we progressively reduced hospitalisation rates in the northern regions until they were comparable to southern Canada. We also varied product uptake for nirsevimab and RSVpreF between 50 and 100%, since information on real-world uptake for these products will not be available until they have been in practice for several years.

Given the uncertainty in the prices of nirsevimab and RSVpreF, we performed a two-way sensitivity analysis where these prices were varied in tandem between $50 and $1000 and the resulting optimal strategy was determined based on the highest net monetary benefit (calculated as incremental benefit multiplied by a WTP threshold of $100,000 per QALY, minus incremental cost). We also ran probabilistic sensitivity analyses in which all parameters were varied simultaneously over 1000 Monte-Carlo simulations.

In addition, we considered three alternative scenarios. First, we dropped the base case assumption of constant product efficacy and permitted the efficacies of nirsevimab, RSVpreF, and palivizumab to wane over the course of the year. This was done by fitting sigmoid decay functions [15, 51] to the mean cumulative efficacy at 150 days and an assumed endpoint of zero efficacy based on product half-lives, where appropriate. Details on the efficacy waning curves can be found in Additional File 1, Supplementary Material: Methods [6, 52]. Second, we ran the analyses from a societal perspective, incorporating out-of-pocket costs and lost wages for families with hospitalised infants [30, 31, 36]. Third, we ran the analyses from an expanded societal perspective with the inclusion of monetary productivity cost and QALY loss associated with infant mortality.

## Results

### Base case analysis

**Fig. 2:**
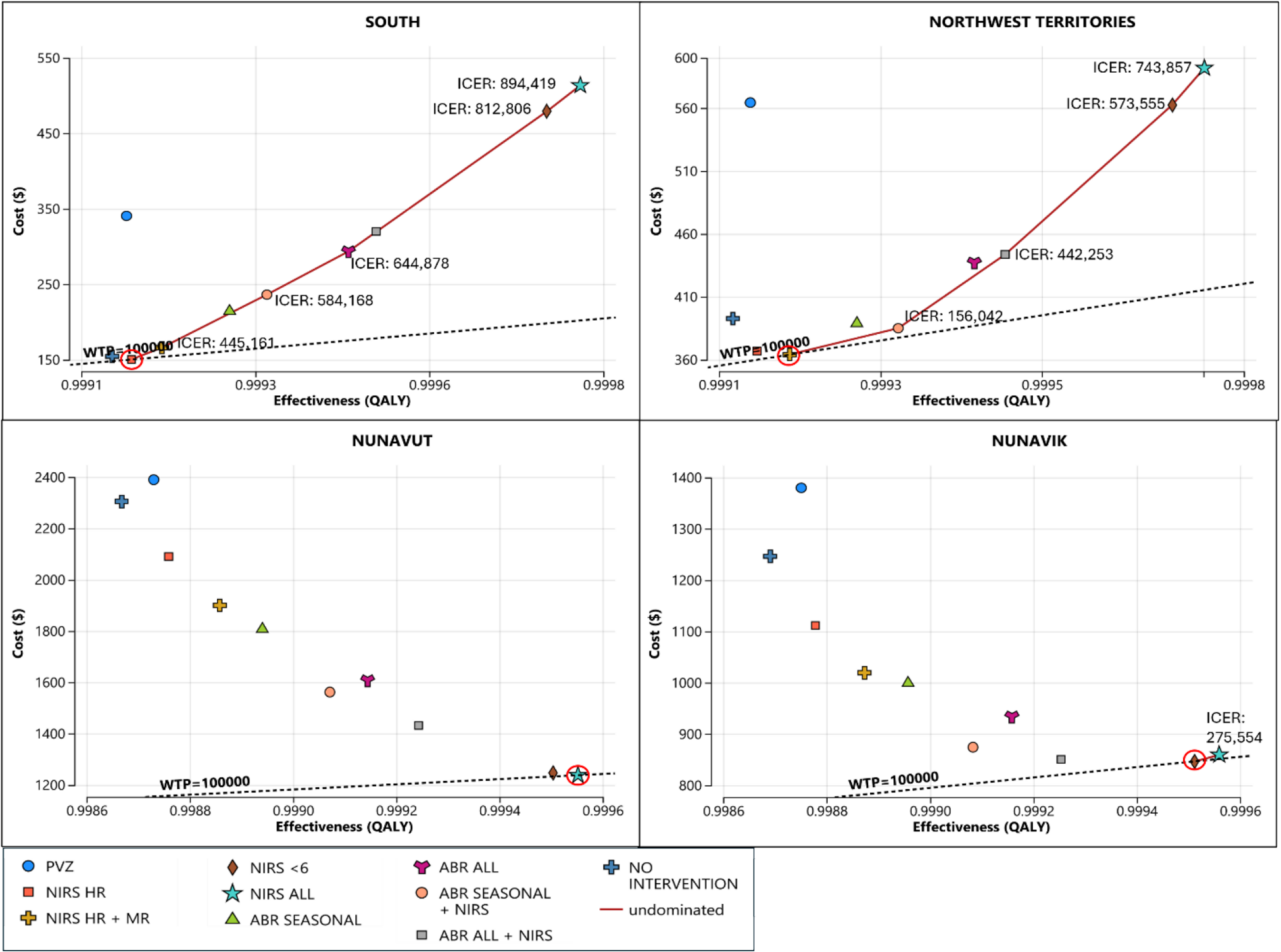
Base case cost-effectiveness results by region. ABR: RSVpreF, HR: high risk, ICER: incremental cost-effectiveness ratio, MR: medium risk, NIRS: nirsevimab, PVZ: palivizumab, QALY: quality-adjusted life year. Costs and QALYs are per infant. ICERs are measured in $/QALY. Red circles indicate the most cost-effective strategy

Base case analysis results are summarised in Fig. 2. In every region, the cheapest strategy is optimal, but which strategy is cheapest depends on the balance between vaccination costs and the cost of treating infections. The cost breakdown for each region is listed in Table 1.

**Table 1:** Cost breakdown by region

Strategy	Vaccination costs per infant ($)	Treatment costs per infant ($)	Total costs per infant ($)
South			
No intervention	0	154	154
PVZ	197	143	341
** NIRS HR**	**12**	**138**	**150**
NIRS HR + MR	40	126	166
NIRS < 6	429	50	479
NIRS ALL	467	46	513
ABR SEASONAL	102	112	214
ABR SEASONAL + NIRS	141	95	236
ABR ALL	204	90	294
ABR ALL + NIRS	243	76	320
Northwest Territories			
No intervention	0	393	393
PVZ	197	367	564
NIRS HR	12	355	367
** NIRS HR + MR**	**40**	**325**	**364**
NIRS < 6	429	134	562
NIRS ALL	467	125	592
ABR SEASONAL	102	287	389
ABR SEASONAL + NIRS	141	244	385
ABR ALL	204	233	437
ABR ALL + NIRS	243	200	444
Nunavut			
No intervention	0	2305	2305
PVZ	237	2153	2390
NIRS HR	14	2075	2090
NIRS HR + MR	52	1848	1900
NIRS < 6	429	818	1247
** NIRS ALL**	**467**	**771**	**1238**
ABR SEASONAL	102	1705	1807
ABR SEASONAL + NIRS	154	1409	1562
ABR ALL	204	1404	1608
ABR ALL + NIRS	256	1176	1432
Nunavik			
No intervention	0	1246	1246
PVZ	234	1146	1380
NIRS HR	14	1097	1112
NIRS HR + MR	51	968	1019
** NIRS < 6**	**429**	**418**	**847**
NIRS ALL	467	393	860
ABR SEASONAL	12	987	999
ABR SEASONAL + NIRS	153	722	874
ABR ALL	204	730	934
ABR ALL + NIRS	255	596	850

*ABR* RSVpreF, *HR* high risk, *MR* medium risk, *NIRS* nirsevimab, *PVZ* palivizumab. Optimal strategy in each region is in bold

In southern Canada, the most cost-effective strategy is to replace palivizumab with nirsevimab administered only to high-risk infants. This strategy saves $4.14 and adds 0.000022 QALY per infant compared to no intervention. None of the expanded coverage strategies were cost-effective under base case prices for nirsevimab and RSVpreF, with ICERs over $440,000 per QALY.

In Nunavut, which has the highest hospitalisation rates and the highest transportation costs in the country, it is most cost-effective to administer nirsevimab to the entire birth cohort. In fact, this strategy is cost-saving relative to all other strategies due to the savings from averted infections. In this region, universal nirsevimab administration saves $1067.03 and adds 0.000884 QALY per infant compared to no intervention.

In Nunavik, which has similar hospitalisation rates as Nunavut but lower transportation costs, it is most cost-effective to administer nirsevimab to all infants under 6 months. This strategy saves $399.61 and adds 0.000821 QALY per infant compared to no intervention. Any strategy of less extensive coverage is dominated—these strategies are more expensive and produce fewer QALYs. The only more extensive strategy—administering nirsevimab universally—is not cost-effective, with an ICER of $275,554 per QALY.

In the Northwest Territories, which has hospitalisation rates and transportation costs that lie between those in southern Canada and the other northern regions, it is most cost-effective to administer nirsevimab to high-risk and medium-risk infants, i.e., all infants born at less than 37 wGA or infants with CHD or CLD. This strategy saves $28.68 and adds 0.00007 QALY per infant compared to no intervention. More restrictive strategies are dominated, and more extensive strategies are either dominated or not cost-effective, with ICERs over $150,000 per QALY.

**Fig. 3:**
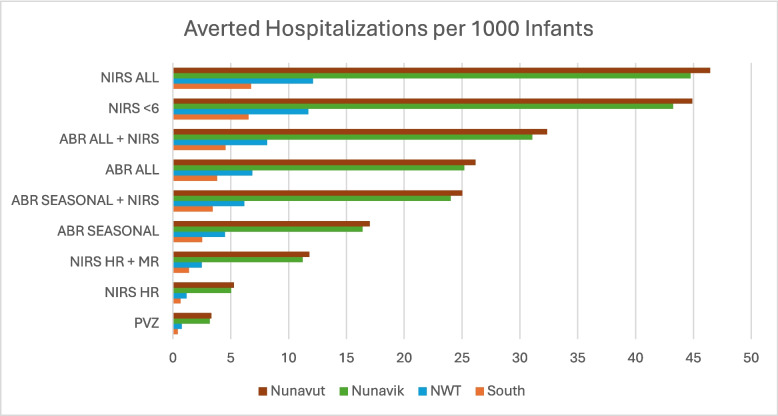
Number of averted hospitalisations with each strategy, by region. ABR, RSVpreF; HR, high risk; MR, medium risk; NIRS, nirsevimab; PVZ, palivizumab

Averted hospitalisations for each strategy are shown in Fig. 3. We calculated the cost of averting one hospitalisation for each new strategy compared to no intervention; these results are listed in Table 2. In Nunavut and Nunavik, every new strategy is cheaper than no intervention; spending money on prophylactics saves money overall. In the Northwest Territories and in southern Canada, nirsevimab and RSVpreF strategies based on selective eligibility criteria are cost-saving compared to no intervention, while administering prophylactics more broadly requires spending between $6247 and $53,113 to prevent one hospitalisation. For context, hospitalising an infant for RSV can cost anywhere between $5000 and $40,000, depending on the length of stay and whether ICU admission is required.

**Table 2:** Cost per one averted hospitalisation, compared to no intervention

	South	NWT	Nunavut	Nunavik
NIRS HR	Cost saving	Cost saving	Cost saving	Cost saving
NIRS HR + MR	$8139	Cost saving	Cost saving	Cost saving
ABR SEASONAL	$23,896	Cost saving	Cost saving	Cost saving
ABR SEASONAL + NIRS	$23,790	Cost saving	Cost saving	Cost saving
ABR ALL	$36,396	$6511	Cost saving	Cost saving
ABR ALL + NIRS	$36,378	$6247	Cost saving	Cost saving
NIRS < 6	$49,683	$14,486	Cost saving	Cost saving
NIRS ALL	$53,113	$16,441	Cost saving	Cost saving

*ABR* RSVpreF, *HR* high risk, *MR* medium risk, *NIRS* nirsevimab, *PVZ* palivizumab

### Sensitivity and scenario analyses

**Fig. 4:**
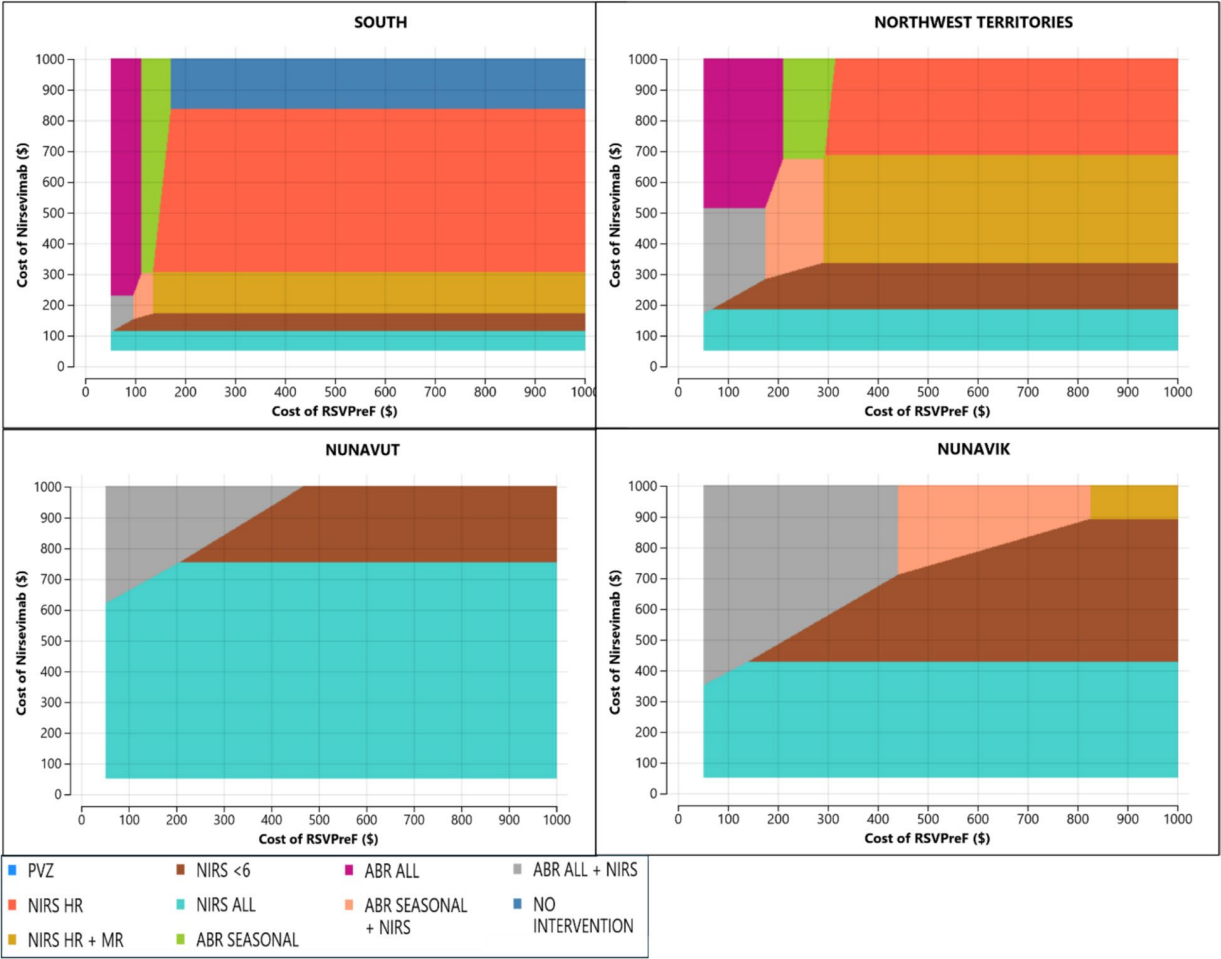
Optimal strategy with varying product prices. ABR, RSVpreF; HR, high risk; MR, medium risk; NIRS, nirsevimab; PVZ, palivizumab

Results in all regions are sensitive to the prices per dose of nirsevimab and RSVpreF. Figure 4 depicts how the optimal strategy (based on the highest NMB) varies with alternative prices of nirsevimab and RSVpreF, based on two-way sensitivity analyses where these prices were varied in tandem. Tables S2 and S3 (Additional File 1) specify the threshold prices at which increased offering of nirsevimab or RSVpreF becomes cost-effective for each region, based on one-way sensitivity analyses of the price of nirsevimab (for nirsevimab-only strategies) and the price of RSVpreF (for RSVpreF-only strategies). Nirsevimab would need to be less than $306 per dose before expanding coverage beyond palivizumab-eligible infants becomes cost-effective nationwide.

In southern Canada, the most cost-effective strategy is impacted only by changing product prices. In the northern regions, the optimal strategy also depends on hospitalisation rates. We assumed successively lower hospitalisation rates in the north until they were comparable to southern Canada. For the Northwest Territories and Nunavik, as hospitalisation rates approach southern levels, the optimal strategy aligns with that of southern Canada (NIRS HR). In Nunavut, even if hospitalisation rates are reduced to southern levels, an expanded coverage strategy (NIRS HR + MR) is still more cost-effective due to the high transportation costs for this region.

In the base case analysis, product uptake was set to 85% for nirsevimab and 65% for RSVpreF, and a nirsevimab-only strategy was optimal for every region. We varied uptake for each product between 50 and 100%, while holding uptake for the alternative product constant at base case levels, and found that if nirsevimab uptake is reduced to 61%, 65%, and 79% for the Northwest Territories, Nunavut, and Nunavik respectively, an RSVpreF-nirsevimab combination strategy is preferred. Details on the thresholds for hospitalisation rates and product uptake can be found in Tables S4 and S5 (Additional file 1).

**Fig. 5:**
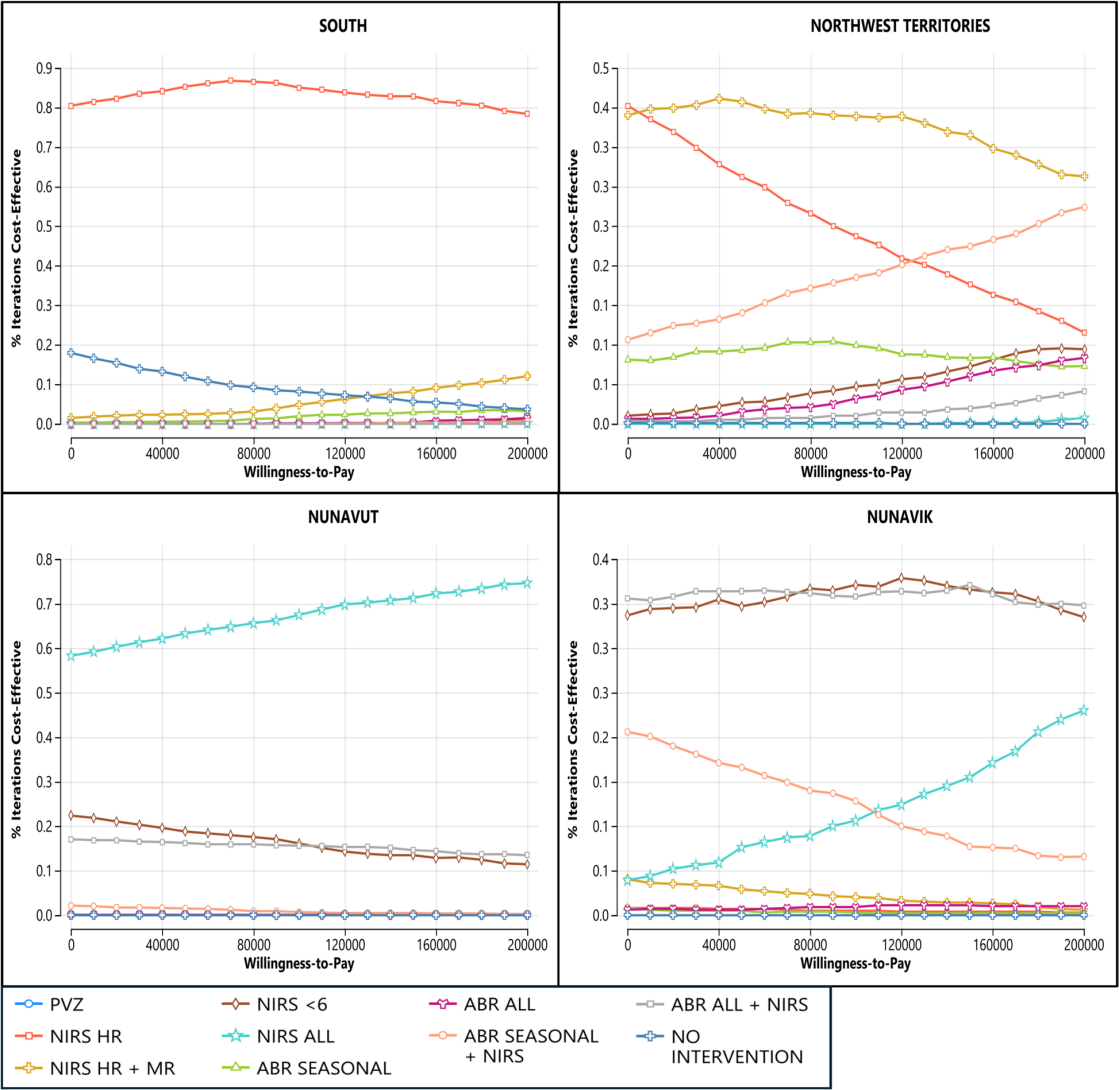
Cost-effectiveness acceptability curves. ABR, RSVpreF; HR, high risk; MR, medium risk; NIRS, nirsevimab; PVZ, palivizumab

Varying all parameters together through probabilistic sensitivity analyses produces the cost-effectiveness acceptability curves found in Fig. 5. In southern Canada and Nunavut, the base case optimal strategy is the most cost-effective strategy in 85% and 68% of iterations at a WTP threshold of $100,000 per QALY, respectively. The Northwest Territories and Nunavik are middle-ground regions for hospitalisation rates and transportation costs, and the optimal strategy for these regions is less certain. In the Northwest Territories, the base case optimal strategy is most cost-effective in 39% of iterations at a WTP threshold of $100,000 per QALY. In Nunavik, two strategies have an approximately equal possibility of being most cost-effective across all WTP thresholds: NIRS < 6 (the base case optimal strategy, most cost-effective in 37% of iterations at $100,000/QALY WTP) and ABR ALL + NIRS. The relative cost-effectiveness of these strategies is sensitive to product price, efficacy, and uptake, and choosing between them will depend on real-world data for these parameters.

Finally, we allowed the efficacy of nirsevimab, RSVpreF, and palivizumab to wane over the course of the year, and we repeated the analysis using two societal perspectives. Full cost-effectiveness results for these scenarios can be found in Figs. S2–S4 (Additional File 1). Neither the waning vaccine scenario nor the first societal perspective scenario resulted in any changes to the overall conclusions. However, incorporating the monetary and QALY loss of infant mortality makes universal nirsevimab administration cost-effective in Nunavik (ICER $38,855/QALY) and seasonal RSVpreF administration with nirsevimab top-ups cost-effective in the Northwest Territories (ICER $14,129/QALY). Importantly, even with this perspective, expanding prophylactic coverage beyond high-risk infants is not cost-effective in southern Canada.

## Discussion

This study evaluated the cost-effectiveness of strategies for preventing RSV disease in infants across Canada, with differentiation between southern Canada and three regions of northern Canada. Replacing palivizumab with nirsevimab—a more effective, less costly option—will be cost-saving nationwide, but the cost-effectiveness of expanding coverage beyond high risk palivizumab-eligible infants depends on product prices and regional variation in hospitalisation rates and resource use. Universal nirsevimab immunisation is the most cost-effective strategy in Nunavut as long as nirsevimab is under $753 per dose, while in southern Canada, nirsevimab would need to be under $112 per dose for universal administration to be cost-effective. For the Northwest Territories and Nunavik, the price per dose would have to be under $183 and $428, respectively.

With RSVpreF, expanded coverage beyond palivizumab-eligible infants is necessitated by its administration to pregnant mothers; we cannot determine which infants will be high risk entirely in advance. In every region, RSVpreF-only strategies were out-ranked by nirsevimab or nirsevimab-RSVpreF combination strategies.

Our model identified a different optimal strategy for every region. This highlights the importance of incorporating regional risk differentiation into cost-effectiveness estimates for RSV prophylactics in Canada. Palivizumab guidelines form a precedent for integrating regional risk into policy; they include specifications for infants living in remote northern communities. Our study is the first cost-effectiveness analysis of nirsevimab and RSVpreF to include detailed differentiation of Canadian northern regions and can help inform policy for these new prophylactics.

Our study is also the first to include seasonal RSVpreF strategies in its analysis. The PSA curves in Fig. 5 and the results in Fig. 2 show that the relative cost-effectiveness of seasonal versus year-round RSVpreF administration depends on region. In southern Canada and the Northwest Territories, seasonal administration is more cost-effective, in line with current U.S. recommendations. In the higher-risk regions of Nunavut and Nunavik, year-round administration is more likely to be cost-effective.

Our results are in line with other Canadian studies [15, 16, 53]. There is consensus that nirsevimab strategies are more cost-effective than RSVpreF strategies, although nirsevimab-RSVpreF combination strategies can be cost-effective in some situations, and that universal nirsevimab administration is unlikely to be cost-effective country-wide unless the price per dose is sufficiently low. This threshold price for nirsevimab varies between studies depending on comparator strategy and WTP threshold, but ranges between $110 and $290. Our ICERs are higher (and threshold prices lower) than other Canadian studies, reflecting our more conservative approach to included parameters and parameter ranges. Most infants will recover from RSV infection with minimal to no medical intervention. The pricing and coverage of these new prophylactics should reflect this reality.

Our study has several limitations. We did not include any long-term sequelae or healthcare resource use or QALY loss beyond the acute infection. Although there is some evidence that healthcare costs may be elevated post-infection [54] and that RSV infection in infanthood is associated with subsequent wheezing [55], there is uncertainty around the causality of long-term effects and no evidence yet on what impact the new prophylactics may have beyond the acute infection.

Without data for outpatient visit rates in northern Canada, we assumed rates equal to southern Canada; in reality, they may be higher (due to more infections) or lower (due to more serious infections). Similarly, although it is conceivable that hospitalisation outcomes (ICU admissions, mortality) may be worse in the north, where resources are limited and treatment may be delayed, in the absence of data, we assumed equal hospitalisation outcomes between regions. The structure of our model divided resource use into outpatient or inpatient pathways and did not account for the realistic case that a patient could use both and accrue both costs. This may make our results more conservative. Similarly, the risk group division in our model separated infants with CHD from preterm infants and did not include infants with both risk factors. We did not consider other risk groups, such as immunocompromised children or children with Down Syndrome or cystic fibrosis.

Another limitation is that the palivizumab strategy used in the model is only an approximation of current Canadian guidelines. We did not disaggregate preterm infants born before 32 wGA and modelled administration of palivizumab to all infants born before this point. NACI recommends palivizumab only for infants born before 30 wGA in the absence of other risk factors. As a result, our model may have underestimated the cost-effectiveness of the current palivizumab administration, but this is unlikely to have affected its ranking among other strategies.

Finally, variation in the uptake of nirsevimab or RSVpreF impacted the results of our model in northern regions. This is important because the actual future uptake of these products is unknown. Positive receptivity to prophylactics is not a guarantee in an era of increasing vaccine hesitancy. There is precedent for sub-optimal roll-out of RSV prevention efforts in northern Canada. A 2017 pilot program in Nunavik for expanded palivizumab eligibility provoked concerns in healthcare workers regarding the feasibility and workload of the program and, importantly, ethical issues regarding involvement and communication with the Inuit population [56]. This study evaluates the potential for these products to be cost-effective; their real-world influence will be dependent on their careful and sensitive integration into practice.

## Conclusions

Replacing palivizumab with nirsevimab for the prevention of RSV disease in infants in Canada will be cost-saving nationwide. The cost-effectiveness of expanding coverage beyond palivizumab-eligible high-risk infants depends on the price per dose of nirsevimab and regional risk and resource use variation. In southern Canada, direct replacement of palivizumab with nirsevimab to only high-risk infants is most cost-effective unless nirsevimab is less than $306 per dose. In contrast, expanded coverage will be cost-effective in Nunavut and Nunavik at any likely price for nirsevimab, and in the Northwest Territories, expanded coverage will be cost-effective at prices lower than $685 per dose. If uptake or supply of nirsevimab is low, RSVpreF-nirsevimab combination strategies could provide a cost-effective alternative. Canadian policy should account for regional differences in RSV rates and healthcare resource use.

## Supplementary Information

Additional file 1: Tables S1-S5 and Figures S1-S4. Table S1. Model inputs. Table S2. Optimal nirsevimab coverage with decreasing price per dose. Table S3. Optimal RSVpreF coverage with decreasing price per dose. Table S4. Optimal strategy with varying hospitalisation rates. Table S5. Optimal strategy with varying product uptake. Figure S1. Efficacy sigmoid decay functions. Figure S2. Cost-effectiveness results with waning vaccine efficacy. Figure S3. Cost-effectiveness results from societal perspective. Figure S4. Cost-effectiveness results from expanded societal perspective.

## Data Availability

The data generated and/or analysed during this study are predominantly included in the main article and its supplementary file. Any additional data are available from the corresponding author on reasonable request.

## Abbreviations

ABR: RSVpreF
CDC: Centers for Disease Control and Prevention
CHD: Congenital heart disease
CLD: Chronic lung disease of prematurity
FDA: Food and Drug Administration
ICER: Incremental cost-effectiveness ratio
LRTI: Lower respiratory tract infection
MA: Medically attended
NACI: National Advisory Council on Immunization
NIRS: Nirsevimab
NMB: Net monetary benefit
NWT: Northwest Territories
PVZ: Palivizumab
QALY: Quality-adjusted life year
RSV: Respiratory syncytial virus
wGA: Weeks of gestational age
WTP: Willingness-to-pay

## References

[1] Glezen WP, Taber LH, Frank AL, Kasel JA. Risk of primary infection and reinfection with respiratory syncytial virus. Am J Dis Child. 1986;140(6):543–6. 3706232 10.1001/archpedi.1986.02140200053026

[2] Li Y, Wang X, Blau DM, Caballero MT, Feikin DR, Gill CJ, et al. Global, regional, and national disease burden estimates of acute lower respiratory infections due to respiratory syncytial virus in children younger than 5 years in 2019: a systematic analysis. Lancet. 2022;399(10340):2047–64. 35598608 10.1016/S0140-6736(22)00478-0 PMC7613574

[3] National Advisory Committee on Immunization. Recommended use of palivizumab to reduce complications of respiratory syncytial virus infection in infants. Ottawa: Public Health Agency of Canada = Agence de la santé publique du Canada; 2022. 10.14745/ccdr.v48i78a08 PMC10324882 37426290

[4] Provinces spent $43M on preemie drug experts say can be made for a fraction of the cost. CBC News; 2017. Available from: https://www.cbc.ca/news/health/rsv-drug-synagis-palivizumab-premature-infants-abbvie-provinces-health-care-1.4056823.

[5] Griffin MP, Yuan Y, Takas T, Domachowske JB, Madhi SA, Manzoni P, et al. Single-dose nirsevimab for prevention of RSV in preterm infants. N Engl J Med. 2020;383(5):415–25. 32726528 10.1056/NEJMoa1913556

[6] Hammitt LL, Dagan R, Yuan Y, Baca Cots M, Bosheva M, Madhi SA, et al. Nirsevimab for prevention of RSV in healthy late-preterm and term infants. N Engl J Med. 2022;386(9):837–46. 35235726 10.1056/NEJMoa2110275

[7] Kampmann B, Madhi SA, Munjal I, Simões EAF, Pahud BA, Llapur C, et al. Bivalent prefusion F vaccine in pregnancy to prevent RSV illness in infants. N Engl J Med. 2023;388(16):1451–64. 37018474 10.1056/NEJMoa2216480

[8] VFC | Current CDC Vaccine price list | CDC. 2024. Available from: https://www.cdc.gov/vaccines/programs/vfc/awardees/vaccine-management/price-list/index.html.

[9] Centers for Disease Control and Prevention. RSV (Respiratory Syncytial Virus) immunizations | CDC. 2024. Available from: https://www.cdc.gov/vaccines/vpd/rsv/index.html.

[10] National Advisory Committee on Immunization. Respiratory syncytial virus (RSV): Canadian immunization guide. 2023. Available from: https://www.canada.ca/en/public-health/services/publications/healthy-living/canadian-immunization-guide-part-4-active-vaccines/respiratory-syncytial-virus.html.

[11] Banerji A, Panzov V, Young M, Robinson J, Lee B, Moraes T, et al. Hospital admissions for lower respiratory tract infections among infants in the Canadian arctic: a cohort study. CMAJ Open. 2016;4(4):E615–22. 28018874 10.9778/cmajo.20150051 PMC5173479

[12] Buchan SA, Chung H, To T, Daneman N, Guttmann A, Kwong JC, et al. Estimating the incidence of first RSV hospitalization in children born in Ontario, Canada. J Pediatr Infect Dis Soc. 2023;12(7):421–30. 10.1093/jpids/piad045 PMC10389057 37335754

[13] Banerji A, Greenberg D, White LF, Macdonald WA, Saxton A, Thomas E, et al. Risk factors and viruses associated with hospitalization due to lower respiratory tract infections in Canadian inuit children: a case-control study. Pediatr Infect Dis J. 2009;28(8):697–701. 19461554 10.1097/INF.0b013e31819f1f89

[14] Young M, Kandola K, Mitchell R, Leamon A. Hospital admission rates for lower respiratory tract infections in infants in the Northwest Territories and the Kitikmeot region of Nunavut between 2000 and 2004. Paediatr Child Health. 2007;12(7):563–6. 19030426 PMC2528776

[15] Shoukat A, Abdollahi E, Galvani AP, Halperin SA, Langley JM, Moghadas SM. Cost-effectiveness analysis of nirsevimab and maternal RSVpreF vaccine strategies for prevention of Respiratory Syncytial Virus disease among infants in Canada: a simulation study. Lancet Reg Health Am. 2023;28:100629. 38026446 10.1016/j.lana.2023.100629 PMC10663690

[16] Gebretekle GB, Yeung MW, Ximenes R, Cernat A, Simmons AE, Killikelly A, et al. Cost-effectiveness of RSVpreF vaccine and nirsevimab for the prevention of respiratory syncytial virus disease in Canadian infants. Vaccine. 2024;42(21):126164. 39079810 10.1016/j.vaccine.2024.126164

[17] Hall CB, Weinberg GA, Iwane MK, Blumkin AK, Edwards KM, Staat MA, et al. The burden of respiratory syncytial virus infection in young children. N Engl J Med. 2009;360(6):588–98. 19196675 10.1056/NEJMoa0804877 PMC4829966

[18] Gilca R, Billard MN, Rochette M, Papenburg J, Zafack J, Charest H, et al. Evaluation of new palivizumab immunoprophylaxis recommendations in Nunavik infants: results for 2014 to 2017. Quebec City: Institut national de sante publique; 2018.

[19] Lively JY, Curns AT, Weinberg GA, Edwards KM, Staat MA, Prill MM, et al. Respiratory syncytial virus-associated outpatient visits among children younger than 24 months. J Pediatr Infect Dis Soc. 2019;8(3):284–6. 10.1093/jpids/piz011 30840770

[20] Arriola CS, Kim L, Langley G, Anderson EJ, Openo K, Martin AM, et al. Estimated burden of community-onset respiratory syncytial virus-associated hospitalizations among children aged <2 years in the United States, 2014–15. J Pediatr Infect Dis Soc. 2020;9(5):587–95. 10.1093/jpids/piz087 PMC7107566 31868913

[21] Bourdeau M, Vadlamudi NK, Bastien N, Embree J, Halperin SA, Jadavji T, et al. Pediatric RSV-associated hospitalizations before and during the COVID-19 pandemic. JAMA Netw Open. 2023;6(10): e2336863. 37792376 10.1001/jamanetworkopen.2023.36863 PMC10551765

[22] Hall CB, Weinberg GA, Blumkin AK, Edwards KM, Staat MA, Schultz AF, et al. Respiratory syncytial virus-associated hospitalizations among children less than 24 months of age. Pediatrics. 2013;132(2):e341–8. 23878043 10.1542/peds.2013-0303

[23] Wong K, Robinson JL, Hawkes MT. Risk of repeated admissions for respiratory syncytial virus in a cohort of >10 000 hospitalized children. J Pediatr Infect Dis Soc. 2021;10(3):352–8. 10.1093/jpids/piaa077 32706370

[24] Tam J, Papenburg J, Fanella S, Asner S, Barton M, Bergeron C, et al. Pediatric investigators collaborative network on infections in Canada study of respiratory syncytial virus–associated deaths in pediatric patients in Canada, 2003–2013. Clin Infect Dis. 2019;68(1):113–9. 29788036 10.1093/cid/ciy413 PMC7108116

[25] OHIP schedule of benefits and fees | ontario.ca. Available from: http://www.ontario.ca/page/ohip-schedule-benefits-and-fees.

[26] Canadian Institute for Health Information. Hospital spending: focus on the emergency department. Ottawa: CIHI; 2020.

[27] Lanctôt KL, Masoud ST, Paes BA, Tarride JE, Chiu A, Hui C, et al. The cost-effectiveness of palivizumab for respiratory syncytial virus prophylaxis in premature infants with a gestational age of 32–35 weeks: a Canadian-based analysis. Curr Med Res Opin. 2008;24(11):3223–37. 18928643 10.1185/03007990802484234

[28] Chu PY, Hornik CP, Li JS, Campbell MJ, Hill KD. Respiratory syncytial virus hospitalisation trends in children with haemodynamically significant heart disease, 1997–2012. Cardiol Young. 2017;27(1):16–25. 27161255 10.1017/S1047951116000470

[29] Smart KA, Paes BA, Lanctôt KL. Changing costs and the impact on RSV prophylaxis. J Med Econ. 2010;13(4):705–8. 21087075 10.3111/13696998.2010.535577

[30] Mitchell I, Defoy I, Grubb E. Burden of respiratory syncytial virus hospitalizations in Canada. Can Respir J. 2017;2017:1–9. 10.1155/2017/4521302 PMC5697123 29311757

[31] Statistics Canada. Employee wages by industry, annual. 2018. Available from: https://www150.statcan.gc.ca/t1/tbl1/en/tv.action?pid=1410006401.

[32] Statistics Canada. Income explorer, 2021 census. 2022. Available from: https://www12.statcan.gc.ca/census-recensement/2021/dp-pd/dv-vd/income-revenu/index-en.html.

[33] Van Den Berg B. SF-6D population norms. Health Econ. 2012;21(12):1508–12. 22250070 10.1002/hec.1823

[34] Roy LM. Deriving health utility weights for infants with Respiratory Syncytial Virus (RSV). University of British Columbia; 2013. Available from: https://open.library.ubc.ca/soa/cIRcle/collections/ubctheses/24/items/1.0074259.

[35] Okiro EA, White LJ, Ngama M, Cane PA, Medley GF, Nokes DJ. Duration of shedding of respiratory syncytial virus in a community study of Kenyan children. BMC Infect Dis. 2010;10(1):15. 20096106 10.1186/1471-2334-10-15 PMC2822777

[36] Banerji A, Ng K, Moraes TJ, Panzov V, Robinson J, Lee BE. Cost-effectiveness of palivizumab compared to no prophylaxis in term infants residing in the Canadian Arctic. CMAJ Open. 2016;4(4):E623–33. 28443266 10.9778/cmajo.20150052 PMC5396468

[37] Garegnani L, Styrmisdóttir L, Roson Rodriguez P, Escobar Liquitay CM, Esteban I, Franco JV. Palivizumab for preventing severe respiratory syncytial virus (RSV) infection in children. Cochrane Acute Respiratory Infections Group, editor. Cochrane Database Syst Rev. 2021;2021(11). Available from: http://doi.wiley.com/10.1002/14651858.CD013757.pub2. 10.1002/14651858.CD013757.pub2 PMC8594174 34783356

[38] Andabaka T, Nickerson JW, Rojas-Reyes MX, Rueda JD, Bacic Vrca V, Barsic B. Monoclonal antibody for reducing the risk of respiratory syncytial virus infection in children. Cochrane Acute Respiratory Infections Group, editor. Cochrane Database Syst Rev. 2013. Available from: https://doi.wiley.com/10.1002/14651858.CD006602.pub4. 10.1002/14651858.CD006602.pub4 23633336

[39] Mitchell I, Li A, Bjornson CL, Lanctot KL, Paes BA, the CARESS investigators. Respiratory syncytial virus immunoprophylaxis with palivizumab: 12-year observational study of usage and outcomes in Canada. Am J Perinatol. 2022;39(15):1668–77. 33657636 10.1055/s-0041-1725146 PMC9643049

[40] Simões EAF, Madhi SA, Muller WJ, Atanasova V, Bosheva M, Cabañas F, et al. Efficacy of nirsevimab against respiratory syncytial virus lower respiratory tract infections in preterm and term infants, and pharmacokinetic extrapolation to infants with congenital heart disease and chronic lung disease: a pooled analysis of randomised controlled trials. Lancet Child Adolesc Health. 2023;7(3):180–9. 36634694 10.1016/S2352-4642(22)00321-2 PMC9940918

[41] Public Health Agency of Canada. Highlights from the 2021 childhood National Immunization Coverage Survey (cNICS). 2023. Available from: https://www.canada.ca/en/public-health/services/immunization-vaccines/vaccination-coverage/2021-highlights-childhood-national-immunization-coverage-survey.html.

[42] Statistics Canada. The daily — recommended vaccines received during pregnancy, 2021. 2022. Available from: https://www150.statcan.gc.ca/n1/daily-quotidien/221213/dq221213a-eng.htm.

[43] Shah PS, Ye XY, Yang J, Campitelli MA. Preterm birth and stillbirth rates during the COVID-19 pandemic: a population-based cohort study. Can Med Assoc J. 2021;193(30):E1164–72. 34344771 10.1503/cmaj.210081 PMC8354648

[44] Canadian Institute for Health Information. Too early, too small: a profile of small babies across Canada. Ottawa: CIHI; 2009.

[45] Van Wagner V, Osepchook C, Harney E, Crosbie C, Tulugak M. Remote midwifery in Nunavik, Québec, Canada: outcomes of perinatal care for the Inuulitsivik health centre, 2000–2007. Birth. 2012;39(3):230–7. 23281905 10.1111/j.1523-536X.2012.00552.x

[46] Beltempo M, Shah PS, Yoon E, Goswami N. Annual report: 2022. Toronto: Canadian Neonatal network; 2022. p. 1–148.

[47] Liu S, Joseph KS, Luo W, León JA, Lisonkova S, Van den Hof M, et al. Effect of folic acid food fortification in Canada on congenital heart disease subtypes. Circulation. 2016;134(9):647–55. 27572879 10.1161/CIRCULATIONAHA.116.022126 PMC4998126

[48] Gilca R, Billard MN, Zafack J, Papenburg J, Boucher FD, Charest H, et al. Effectiveness of palivizumab immunoprophylaxis to prevent respiratory syncytial virus hospitalizations in healthy full-term <6-month-old infants from the circumpolar region of Nunavik, Quebec. Canada Prev Med Rep. 2020;20:101180. 32953425 10.1016/j.pmedr.2020.101180 PMC7484550

[49] Stevens TP, Sinkin RA, Hall CB, Maniscalco WM, McConnochie KM. Respiratory syncytial virus and premature infants born at 32 weeks’ gestation or earlier: hospitalization and economic implications of prophylaxis. Arch Pediatr Adolesc Med. 2000;154(1):55–61. 10632251

[50] Hodgson D, Atkins KE, Baguelin M, Panovska-Griffiths J, Thorrington D, van Hoek AJ, et al. Estimates for quality of life loss due to respiratory syncytial virus. Influenza Other Respir Viruses. 2020;14(1):19–27. 31625688 10.1111/irv.12686 PMC6928035

[51] Hutton DW. Economic analysis of nirsevimab in pediatric populations. Proceedings of the Advisory Committee on Immunization Practices (ACIP) Meeting; 2023 Feb 22-24. Atlanta: 2023. Available from: https://stacks.cdc.gov/view/cdc/125142.

[52] Griffin MP, Khan AA, Esser MT, Jensen K, Takas T, Kankam MK, et al. Safety, tolerability, and pharmacokinetics of MEDI8897, the respiratory syncytial virus prefusion F-targeting monoclonal antibody with an extended half-life. Healthy Adults Antimicrob Agents Chemother. 2017;61(3):e01714-e1716. 27956428 10.1128/AAC.01714-16 PMC5328523

[53] Nourbakhsh S, Shoukat A, Zhang K, Poliquin G, Halperin D, Sheffield H, et al. Effectiveness and cost-effectiveness of RSV infant and maternal immunization programs: a case study of Nunavik. Canada eClinicalMedicine. 2021;41:101141. 34622186 10.1016/j.eclinm.2021.101141 PMC8479643

[54] Rafferty E, Paulden M, Buchan SA, Robinson JL, Bettinger JA, Kumar M, et al. Evaluating the individual healthcare costs and burden of disease associated with RSV across age groups. Pharmacoeconomics. 2022;40(6):633–45. 35553028 10.1007/s40273-022-01142-w PMC9130187

[55] Blanken MO, Rovers MM, Molenaar JM, Winkler-Seinstra PL, Meijer A, Kimpen JLL, et al. Respiratory syncytial virus and recurrent wheeze in healthy preterm infants. N Engl J Med. 2013;368(19):1791–9. 23656644 10.1056/NEJMoa1211917

[56] Lorcy A, Gilca R, Dubé E, Rochette M, De Serres G. Feasibility and ethical issues: experiences and concerns of healthcare workers regarding a new RSV prophylaxis programme in Nunavik, Quebec. Int J Circumpolar Health. 2020;79(1):1742564. 32191589 10.1080/22423982.2020.1742564 PMC7144279

