# Supplementary material for: Cost-effectiveness of nirsevimab and maternal RSVpreF for preventing respiratory syncytial virus disease in infants across Canada
Source: BMC Med. 2025 Feb 21;23:102. doi: 10.1186/s12916-025-03928-z (PMC11846277; doi:10.1186/s12916-025-03928-z)
Supplement: Supplementary file 1 — Additional file 1: Tables S1-S5 and Figures S1-S4. Table S1. Model inputs. Table S2. Optimal nirsevimab coverage with decreasing price per dose. Table S3. Optimal RSVpreF coverage with decreasing price per dose. Table S4. Optimal strategy with varying hospitalisation rates. Table S5. Optimal strategy with varying product uptake. Figure S1. Efficacy sigmoid decay functions. Figure S2. Cost-effectiveness results with waning vaccine efficacy. Figure S3. Cost-effectiveness results from societal perspective. Figure S4. Cost-effectiveness results from expanded societal perspective. [file 12916_2025_3928_MOESM1_ESM.docx]

Additional file 1 for Cost-effectiveness of Nirsevimab and Maternal RSVpreF for Preventing Respiratory Syncytial Virus Disease in Infants Across Canada

Samara Bugden, Shweta Mital, PhD, Hai V. Nguyen, PhD

Contents

Supplementary Material: Methods……………………………………………………… 2

Model Inputs …………………………………………………………………… 2

RSV outcomes …………………………………………………………. 2

Costs and utilities ………………………………………………………. 3

Northern regions ……………………………………………………….. 4

Efficacy estimates ……………………………………………………… 5

Table S1. Model inputs ………………………………………………………… 6

Efficacy Waning ………………………………………………….……………. 9

Figure S1. Efficacy sigmoid decay functions ………………………….. 10

Supplementary Material: Results ..…………………………………………………….. 11

Table S2. Optimal nirsevimab coverage with decreasing price per dose ……… 11

Table S3. Optimal RSVpreF coverage with decreasing price per dose ………. 11

Table S4. Optimal strategy with varying hospitalisation rates ………………… 12

Table S5. Optimal strategy with varying product uptake ……………………… 12

Figure S2. Cost-effectiveness results with waning vaccine efficacy …………… 13

Figure S3. Cost-effectiveness results from societal perspective ……………….. 14

Figure S4. Cost-effectiveness results from expanded societal perspective …….. 15

**Supplementary Material: Methods**

**Model Inputs**

***RSV outcomes***

A full list of model inputs and their ranges can be found in Table S1.

The model tracked medically-attended RSV infection, including primary care visits, emergency department visits, and hospitalisations, a percentage of which require intensive care unit (ICU) admission. The rates and probabilities of all these outcomes are age dependent. RSV rates also fluctuate seasonally. For each monthly birth cohort in the model, a yearly weighted average rate for each outcome was calculated by multiplying the incidence rate at age *j* by the percentage of RSV hospitalisations occurring in calendar month *k* and then summing. In this way, infants who were younger during peak months had a greater yearly risk of medically-attended RSV infection.

20% of RSV hospitalisations occur in infants with prematurity/comorbidity risk factors (11,12,22). Overall hospitalisation incidence rates for infants 0-11 months were reduced by 20% to form a baseline rate for healthy full-term infants. Then, baseline hospitalisation rates were multiplied by incidence rate ratios of 1.99 and 4.26 for premature babies born at 33-36 weeks of gestational age (wGA) and <33 wGA, respectively, while for babies with CHD and CLD they were multiplied by ratios of 4.13 and 4.95 (12). Baseline incidence rates of first hospitalisation were also increased by the probability of readmission for RSV infection within the same year, estimated at 4.49% for low risk infants, 4.74% for preterm infants, 4.98% for infants with CLD, and 5.20% for infants with CHD (23).

The model also included RSV-related mortality. One Canadian study of RSV hospitalisations found a mortality rate of 0.3% but did not specify how many of those deaths were considered to be due to RSV (21). Another study of RSV-associated deaths in Canada found RSV to be a contributing factor or primary cause in 70% of deaths occurring in pediatric RSV hospitalisations (24), so an overall mortality rate of 0.21% was used.

***Costs and utilities***

The base case analysis considered only direct health care costs. Primary care visits and emergency department visits were set to $87.35 (25) and $424.04 (26), respectively. Hospitalisation costs were based on ward ($1384.75) and ICU ($3378.71) per diem costs for a pediatric RSV admission (27) and length of stay (LoS), which varied according to risk group, from an LoS of 3.5 days for low risk infants admitted to a general ward (3), to an LoS of 12.1 days for infants with CHD (28).

In the absence of Canadian drug costing information, drug costs for nirsevimab and RSVpreF were set to $533.29 and $298.70, respectively, based on the United States Center for Disease Control vaccine contract prices (8) plus a $15 administration fee per dose (15), and varied from $50 to $1000 in the sensitivity analyses. The cost of a full course of palivizumab, which must be administered monthly, was set to $9078.95 based on the average number of vials required and the proportion of infants requiring 50 or 100 mg vials (27,29). We did not include any variation between regions in travel costs for obtaining prophylactics, based on the expectation that receipt of prophylactics could be bundled into other care, such as a hospital admission for birth or a public health visit for other infant vaccines, for nirsevimab, or a prenatal visit, for RSVpreF.

The societal perspective analysis included out-of-pocket costs for families and the cost of parents’ missed work when infants are hospitalised with RSV infections. Out-of-pocket costs were based on a study of RSV hospitalisation disease burden in Canada (30) and included transportation to/from the hospital, meals/lodging, child care, and any over-the-counter medication and medical equipment, amounting to $906.26. The cost of missed work was set to $828.69, based on an average of 24.7 hours of missed work (30) and an average hourly wage in Canada of $33.55 (31). We also ran an expanded societal perspective analysis that incorporated the impact of loss of life due to infant mortality. Monetary loss of life was set to $1,098,324, calculated by summing the average income per year over the working years in a lifespan (32), discounted at a rate of 1.5%. QALY loss was set to 38.07, the quality-adjusted life expectancy discounted at 1.5% (33).

Effectiveness was measured in quality-adjusted life years (QALYs). Each level of RSV infection (outpatient, ward hospitalisation, ICU admission) was assigned a disutility (34), and that disutility was applied for the duration of the illness. Illness duration was considered to be either hospital length of stay, or an average duration of 6.16 days (35), whichever was greater. If LoS was less than 6.16 days, outpatient disutility was applied to the difference between 6.16 and LoS. Death was assigned a utility of 0.5 QALY to reflect an average of death occurring midway through the year.

***Northern regions***

Monthly granularity in hospitalisation rates is not available for northern Canada. Instead, incidence rates for infants under one year old were used (15.8, 60.2, and 58.1 per 1000 live births for the Northwest Territories, Nunavut, and Nunavik, respectively) (11) by assuming the age distribution for hospitalisations is equivalent to southern Canada, and by assuming the seasonal distribution follows the same pattern, just shifted by two months to reflect the delayed northern season. Outpatient visit rates and hospitalisation outcomes were kept the same as southern Canada.

We included transportation costs incurred when hospitalising infants from the north, set to $8070.30 for the Northwest Territories, $20,484.91 for Nunavut, and $6529.58 for Nunavik (36), based on the cost of medical air evacuation to a regional or tertiary hospital and an economy return flight.

***Efficacy estimates***

For each product, we used three measures of efficacy: efficacy against medically-attended (MA) RSV infection, efficacy against hospitalisation, and efficacy against ICU admission. Palivizumab efficacies were based on Cochrane reviews (37,38). 83.8% of infants receiving palivizumab obtain all expected doses (39); this number was used to approximate palivizumab uptake. Nirsevimab efficacies were based on pooled trial results (40) of efficacy through 150 days post-dose, with efficacy against “very severe RSV disease” used for ICU admission. Uptake for nirsevimab was set to 85.2%, the average uptake rate for childhood vaccines in Canada in 2021 (41). RSVpreF efficacies through 150 days post-birth were obtained from trial results (7), with efficacy against “medically-attended severe” RSV infection used for ICU admission. RSVpreF uptake was set to 65%, the percentage of women in Canada who receive the recommended pertussis vaccine in their third trimester of pregnancy (42). In the case of combination strategies where some infants received both products, the higher efficacy between the two products was applied.

Table S1. Model inputs

| Parameter | | | Base Case | Range | | Source |
| --- | --- | --- | --- | --- | --- | --- |
|  | | | | (min) | (max) |  |
| **Probabilities** | | | | | | |
| Proportion of births: | | |  |  |  |  |
|  | Preterm 33-37 wGA – South, NWT | | 0.0642 | 0.0482 | 0.0803 | (43) |
|  | Preterm <33 wGA – South, NWT | | 0.0154 | 0.0116 | 0.0193 | (43) |
|  | Preterm 33-37 wGA – Nunavut | | 0.0871 | 0.0653 | 0.1089 | (43,44) |
|  | Preterm <33 wGA – Nunavut | | 0.0209 | 0.0157 | 0.0261 | (43,44) |
|  | Preterm 33-37 wGA – Nunavik | | 0.0855 | 0.0641 | 0.1069 | (43,45) |
|  | Preterm <33 wGA – Nunavik | | 0.0205 | 0.0154 | 0.0256 | (43,45) |
|  | CLD, given preterm <33 wGA | | 0.326 | 0.245 | 0.408 | (46) |
|  | CHD | | 0.0112 | 0.0084 | 0.0140 | (47) |
| Southern hospitalisation rates (/1000): | | |  |  |  | (12) |
|  | 0 months | | 13.50 | 14.27 | 15.10 |  |
|  | 1 month | | 22.63 | 23.64 | 24.70 |  |
|  | 2 months | | 12.30 | 12.82 | 13.38 |  |
|  | 3 months | | 12.30 | 12.82 | 13.38 |  |
|  | 4 months | | 7.51 | 7.92 | 8.36 |  |
|  | 5 months | | 7.51 | 7.92 | 8.36 |  |
|  | 6 months | | 4.85 | 5.11 | 5.40 |  |
|  | 7 months | | 4.85 | 5.11 | 5.40 |  |
|  | 8 months | | 4.85 | 5.11 | 5.40 |  |
|  | 9 months | | 3.20 | 3.42 | 3.66 |  |
|  | 10 months | | 3.20 | 3.42 | 3.66 |  |
|  | 11 months | | 3.20 | 3.42 | 3.66 |  |
| Northern hospitalisation rates (/1000): | | | NWT: | Nunavut: | Nunavik: | (11,48) |
|  | 0-11 months | | 15.8 | 60.2 | 58.1 |  |
| Hospitalisation Incidence Rate Ratios | | |  |  |  | (12) |
|  | Preterm 33-37 wGA | | 1.99 | 1.49 | 2.49 |  |
|  | Preterm <33 wGA | | 4.02 | 3.02 | 5.03 |  |
|  | CLD | | 4.95 | 3.71 | 6.19 |  |
|  | CHD | | 4.13 | 3.10 | 5.16 |  |
| Probability of readmission within the same season | | | |  |  | (23) |
|  | Low risk | | 0.0449 | 0.0337 | 0.0561 |  |
|  | Preterm <37 wGA | | 0.0474 | 0.0356 | 0.0593 |  |
|  | CLD | | 0.0498 | 0.0374 | 0.0623 |  |
|  | CHD | | 0.0520 | 0.0390 | 0.0650 |  |
| Percentage of hospitalisations with ICU admission: low risk, high risk (preterm or with comorbidity) (%) | | | | | | |
|  | 0 months | | 36.9, 50 | 28, 34 | 46, 65 | (20,21) |
|  | 1 month | | 31,50 | 23, 34 | 39, 65 |  |
|  | 2 months | | 23.9, 50 | 18, 34 | 30, 65 |  |
|  | 3 months | | 23.8, 29 | 18, 6 | 30, 49 |  |
|  | 4 months | | 15.1, 29 | 11, 6 | 19, 49 |  |
|  | 5 months | | 21, 19 | 16, 6 | 26, 49 |  |
|  | 6 months | | 19.5, 19 | 15, 7 | 24, 30 |  |
|  | 7 months | | 17.1, 19 | 13, 7 | 21, 30 |  |
|  | 8 months | | 19.6, 19 | 15, 7 | 25, 30 |  |
|  | 9 months | | 14.5, 19 | 11, 7 | 18, 30 |  |
|  | 10 months | | 15, 19 | 11, 7 | 18, 30 |  |
|  | 11 months | | 16, 19 | 12, 7 | 20, 30 |  |
| Primary care visit rates (/1000): | | | |  |  | (19) |
|  | 0 months | | 85.2 | 71 | 99.3 |  |
|  | 1 month | | 187.9 | 156.6 | 219.1 |  |
|  | 2 months | | 234.2 | 195.2 | 273.1 |  |
|  | 3 months | | 232.6 | 194 | 271.3 |  |
|  | 4 months | | 265 | 221 | 3069.1 |  |
|  | 5 months | | 289.2 | 241.1 | 337.2 |  |
|  | 6 months | | 264.7 | 220.7 | 308.7 |  |
|  | 7 months | | 207.2 | 172.8 | 241.7 |  |
|  | 8 months | | 277.8 | 231.7 | 324 |  |
|  | 9 months | | 227.2 | 189.4 | 264.9 |  |
|  | 10 months | | 241.7 | 201.5 | 281.8 |  |
|  | 11 months | | 258.1 | 215.2 | 301 |  |
| Emergency department visit rates (/1000): | | | | |  | (19) |
|  | 0 months | | 19.6 | 16.8 | 22.4 |  |
|  | 1 month | | 64.2 | 54.9 | 73.4 |  |
|  | 2 months | | 72.4 | 62 | 82.9 |  |
|  | 3 months | | 105.2 | 90.1 | 120.4 |  |
|  | 4 months | | 116 | 99.3 | 132.7 |  |
|  | 5 months | | 71.3 | 61.1 | 81.6 |  |
|  | 6 months | | 81.8 | 70.1 | 93.6 |  |
|  | 7 months | | 56.1 | 48 | 64.2 |  |
|  | 8 months | | 55.6 | 47.6 | 63.5 |  |
|  | 9 months | | 55.6 | 47.6 | 63.6 |  |
|  | 10 months | | 40.4 | 31.6 | 46.2 |  |
|  | 11 months | | 55.6 | 47.6 | 63.6 |  |
| RSV mortality (% of hospitalisations): | | | 0.21 | 0.16 | 0.26 | (21,24) |
| Vaccine efficacy against medically attended RSV infection (%): | | | | | | (7,37,38,40) |
|  | Nirsevimab | | 79.5 | 59.6 | 99.4 |  |
|  | RSVpreF | | 52.5 | 39.4 | 65.6 |  |
|  | Palivizumab | | 67.0 | 50.2 | 83.7 |  |
| Vaccine efficacy against hospitalisation: | | | |  |  |  |
|  | Nirsevimab | | 77.3 | 58.0 | 96.6 |  |
|  | RSVpreF | | 56.4 | 42.3 | 70.5 |  |
|  | Palivizumab | | 50.5 | 37.9 | 63.1 |  |
| Vaccine efficacy against ICU admission: | | | | |  |  |
|  | Nirsevimab | | 86.0 | 64.5 | 100.0 |  |
|  | RSVpreF | | 70.9 | 53.2 | 88.6 |  |
|  | Palivizumab | | 50.0 | 37.5 | 62.5 |  |
| Product uptake (%): | | |  |  |  |  |
|  | Nirsevimab | | 85.2 | 50 | 100 | (41) |
|  | RSVpreF | | 65 | 50 | 100 | (42) |
|  | Palivizumab | | 83.8 | 50 | 100 | (39) |
| **Costs (2024 CAD)** | | | | |  |  |
| Cost of primary care visit: | | | 87.35 | 65.51 | 109.19 | (25) |
| Emergency department visit: | | | 424.02 | 318.02 | 530.03 | (25,26) |
| Hospitalisation: | | |  |  |  |  |
|  | Per diem cost | | 1384.75 | 1038.56 | 1730.94 | (27) |
|  | Length of stay (days) | |  |  |  |  |
|  | | Low risk | 3.5 | 2.6 | 4.4 | (3) |
|  | | Preterm 33-37 wGA | 4.5 | 3.8 | 5.6 | (3) |
|  | | Preterm <33 wGA | 7.2 | 5.4 | 9.0 | (3) |
|  | | CLD | 9.4 | 7.1 | 11.8 | (49) |
|  | | CHD | 12.1 | 9.1 | 15.1 | (28) |
| ICU admission: | | |  |  |  |  |
|  | Per diem cost | | 3378.71 | 2534.03 | 4223.39 | (27) |
|  | Length of stay (days) | |  |  |  |  |
|  | | Low risk | 6.2 | 4.7 | 7.8 | (30) |
|  | | Preterm 33-37 wGA | 6.7 | 5.0 | 8.4 | (3) |
|  | | Preterm <33 wGA | 9.0 | 6.8 | 11.3 | (3) |
|  | | CLD | 9.4 | 7.1 | 11.8 | (49) |
|  | | CHD | 12.1 | 9.1 | 15.1 | (28) |
| Medical transport and accommodation for Northern regions: | | | |  |  | (36) |
|  | NWT | | 8070.30 | 6052.73 | 10,087.88 |  |
|  | Nunavut | | 20,484.91 | 15,363.68 | 25,606.14 |  |
|  | Nunavik | | 6529.59 | 4897.19 | 8161.98 |  |
| Out of pocket costs: | | | 906.26 |  |  | (30) |
| Cost of parents’ missed work: | | | 828.69 |  |  | (30,31) |
| Monetary loss of life for infant mortality ($) | | | 1,098,324 |  |  | (32) |
| QALY loss for infant mortality | | | 38.07 |  |  | (33) |
| Product prices (including $15 administration fee per dose): | | |  |  |  |  |
|  | Nirsevimab (per dose) | | 548.29 | 50 | 1000 | (8) |
|  | Maternal RSVpreF (per dose) | | 313.70 | 50 | 1000 | (8) |
|  | Palivizumab (per season) | | 9153.95 |  |  | (27,29) |
| **Disutilities (QALYs lost)** | | | | | | (34,50) |
| Outpatient infection | | | 0.0027 | 0.002025 | 0.003375 |  |
| Hospitalisation | | |  |  |  |  |
|  | Low risk | | 0.0051 | 0.003825 | 0.006375 |  |
|  | Preterm 33-37 wGA | | 0.00578 | 0.004335 | 0.007225 |  |
|  | Preterm <33 wGA | | 0.00809 | 0.006068 | 0.010113 |  |
|  | CLD | | 0.01056 | 0.007920 | 0.001320 |  |
|  | CHD | | 0.01359 | 0.010193 | 0.016988 |  |
| ICU admission | | |  |  |  |  |
|  | Low risk | | 0.01019 | 0.007643 | 0.012738 |  |
|  | Preterm 33-37 wGA | | 0.01101 | 0.008258 | 0.013763 |  |
|  | Preterm <33 wGA | | 0.01479 | 0.011093 | 0.018488 |  |
|  | CLD | | 0.01545 | 0.011588 | 0.019313 |  |
|  | CHD | | 0.01989 | 0.014918 | 0.024863 |  |

**Efficacy Waning**

Efficacies for all products were fitted to sigmoid decay functions (15,51), following the formula:

$$VE\left( t \right)=\frac{C}{1+ae^{-bt}}$$

Where VE(t) is vaccine efficacy at time t (in days), C is the maximum of the function, b is the decay rate, and a is a scaling factor affecting the shape of the curve. We used Excel Solver to fit C, b, and a to available efficacy data, which was:

1. For nirsevimab: mean cumulative efficacy through day 150, and the assumption that efficacy will be 0 by day 360, based on a product half-life of 69 days (6).
2. For RSVpreF: mean cumulative efficacies through days 90, 120, 150, 180, and 360.
3. For palivizumab: mean cumulative efficacy through day 150, and the assumption that efficacy will be 0 by 100 days past last dose, based on a half-life of 20 days (52). We assume 5 doses, each one month apart.

The curves for these efficacy functions are pictured in figure S1.

Figure S1. Efficacy sigmoid decay functions

MA: medically attended, ICU: intensive care unit


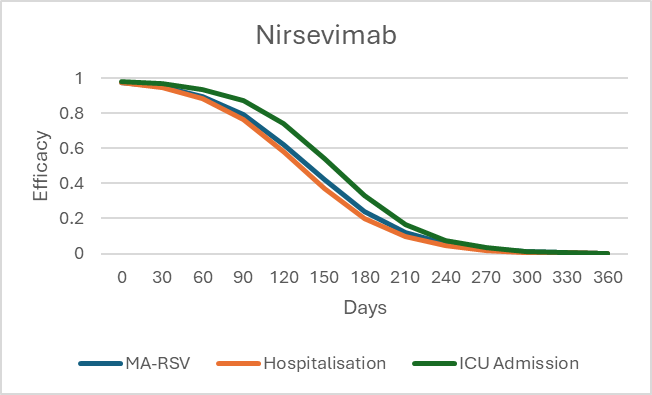

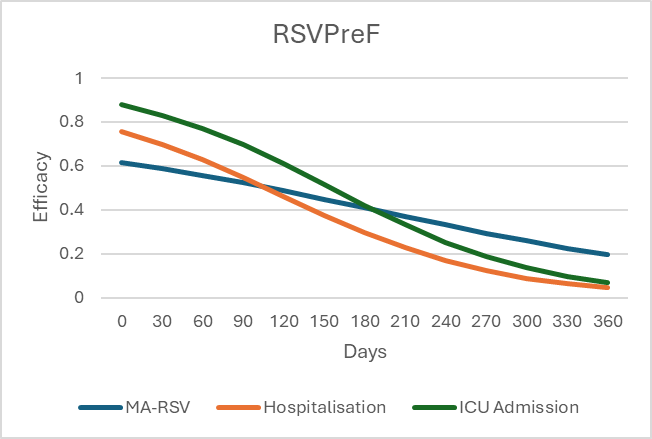

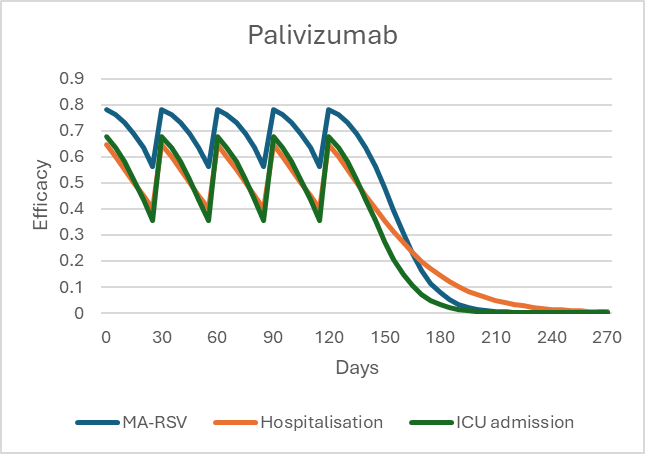


**Supplementary Material: Results**

Table S2. Optimal nirsevimab coverage with decreasing price per dose

| Price Per Dose | Optimal Nirsevimab Coverage | | | |
| --- | --- | --- | --- | --- |
|  | South | Northwest Territories | Nunavut | Nunavik |
| 1000 | $1000: NIRS HR | $1000: NIRS HR | $1000: NIRS <6 | $1000: NIRS HR + MR |
|  |  |  |  |  |
| 900 |  |  |  |  |
|  |  |  |  |  |
| 800 |  |  |  | $891: NIRS <6 |
|  |  |  | $753: NIRS ALL |  |
| 700 |  | $685: NIRS HR + MR |  |  |
|  |  |  |  |  |
| 600 |  |  |  |  |
|  |  |  |  |  |
| 500 |  |  |  |  |
|  |  |  |  | $428: NIRS ALL |
| 400 |  |  |  |  |
|  | $306:NIRS HR + MR | $336: NIRS <6 |  |  |
| 300 |  |  |  |  |
|  |  |  |  |  |
| 200 | $170: NIRS <6 | $183: NIRS ALL |  |  |
|  | $112: NIRS ALL |  |  |  |
| 100 |  |  |  |  |

HR: infants with CHD or CLD, infants born <33 wGA and <6 months old. MR: infants born <37 wGA and <6 months old. NIRS: nirsevimab. Optimal nirsevimab strategy based on highest net monetary benefit, calculated for a WTP threshold of $100,000/QALY. Only nirsevimab-only strategies are included in this analysis; the cost-effectiveness of nirsevimab-RSVpreF combination strategies is also dependent on the price of RSVpreF.

Table S3. Optimal RSVpreF coverage with decreasing price per dose

| Price Per Dose | Optimal RSVpreF Coverage | | | |
| --- | --- | --- | --- | --- |
|  | South | Northwest Territories | Nunavut | Nunavik |
| 1000 | $1000: ABR SEASONAL | $1000: ABR SEASONAL | $988: ABR ALL | $1000: ABR SEASONAL |
| 900 |  |  |  |  |
| 800 |  |  |  |  |
| 700 |  |  |  |  |
| 600 |  |  |  | $575: ABR ALL |
| 500 |  |  |  |  |
| 400 |  |  |  |  |
| 300 |  |  |  |  |
| 200 |  | $209: ABR ALL |  |  |
| 100 | $112: ABR ALL |  |  |  |

Abr: RSVpreF. Optimal RSVpreF strategy based on highest net monetary benefit, calculated for a WTP threshold of $100,000/QALY. Only RSVpreF-only strategies are included in this analysis; the cost-effectiveness of nirsevimab-RSVpreF combination strategies is also dependent on the price of nirsevimab.

Table S4. Optimal strategy with varying hospitalisation rates

| Region | Hospitalisation rate  (/1000, from 0-11 months) | Most cost-effective strategy |
| --- | --- | --- |
| Northwest Territories  (base case hospitalisation rate*: 15.8/1000 infants 0-11 months) | 12.1 – 15.8 | NIRS HR + MR |
|  | <12.1 | NIRS HR |
|  |  |  |
| Nunavut  (base case hospitalisation rate*: 60.2/1000 infants 0-11 months) | 41.7 – 60.2 | NIRS ALL |
|  | 24.1 – 41.7 | NIRS <6 |
|  | 21.1 – 24.1 | ABR ALL + NIRS |
|  | 11.1 – 21.1 | ABR SEASONAL + NIRS |
|  | <11.1 | NIRS HR + MR |
|  |  |  |
| Nunavik  (base case hospitalisation rate*: 58.1/1000 infants 0-11 months) | 48.2 – 58.1 | NIRS <6 |
|  | 38.9 – 48.2 | ABR ALL + NIRS |
|  | 19.1 – 38.9 | ABR SEASONAL + NIRS |
|  | 13.2 – 19.1 | NIRS HR + MR |
|  | <13.2 | NIRS HR |
|  |  |  |
| Southern Canada  (base case hospitalisation rate*: 8.8/1000 infants 0-11 months) | 8.3 – 9.2 | NIRS HR |
|  |  |  |

ABR: RSVpreF, HR: high risk, MR: medium risk, NIRS: nirsevimab. *Hospitalisation rate for healthy full term infants.

Table S5. Optimal strategy with varying product uptake

| Region | Base case strategy  (Nirsevimab uptake 85%, RSVpreF uptake 65%)* | Uptake Change | Alternative strategy: |
| --- | --- | --- | --- |
| Northwest Territories | NIRS HR + MR | Nirsevimab uptake <61% | ABR SEASONAL + NIRS |
| Nunavut | NIRS ALL | Nirsevimab uptake <65%  OR | ABR ALL + NIRS |
|  |  | RSVpreF uptake > 94% | ABR ALL + NIRS |
| Nunavik | NIRS <6 | Nirsevimab uptake <79%  OR | ABR ALL + NIRS |
|  |  | RSVpreF uptake > 74% | ABR ALL + NIRS |

ABR: RSVpreF, HR: high risk, MR: medium risk, NIRS: nirsevimab. Results are from one-way sensitivity analyses where uptake of one product is varied between 50% and 100% while uptake of the other product is held constant at the base case value.

Figure S2. Cost-effectiveness results with waning vaccine efficacy


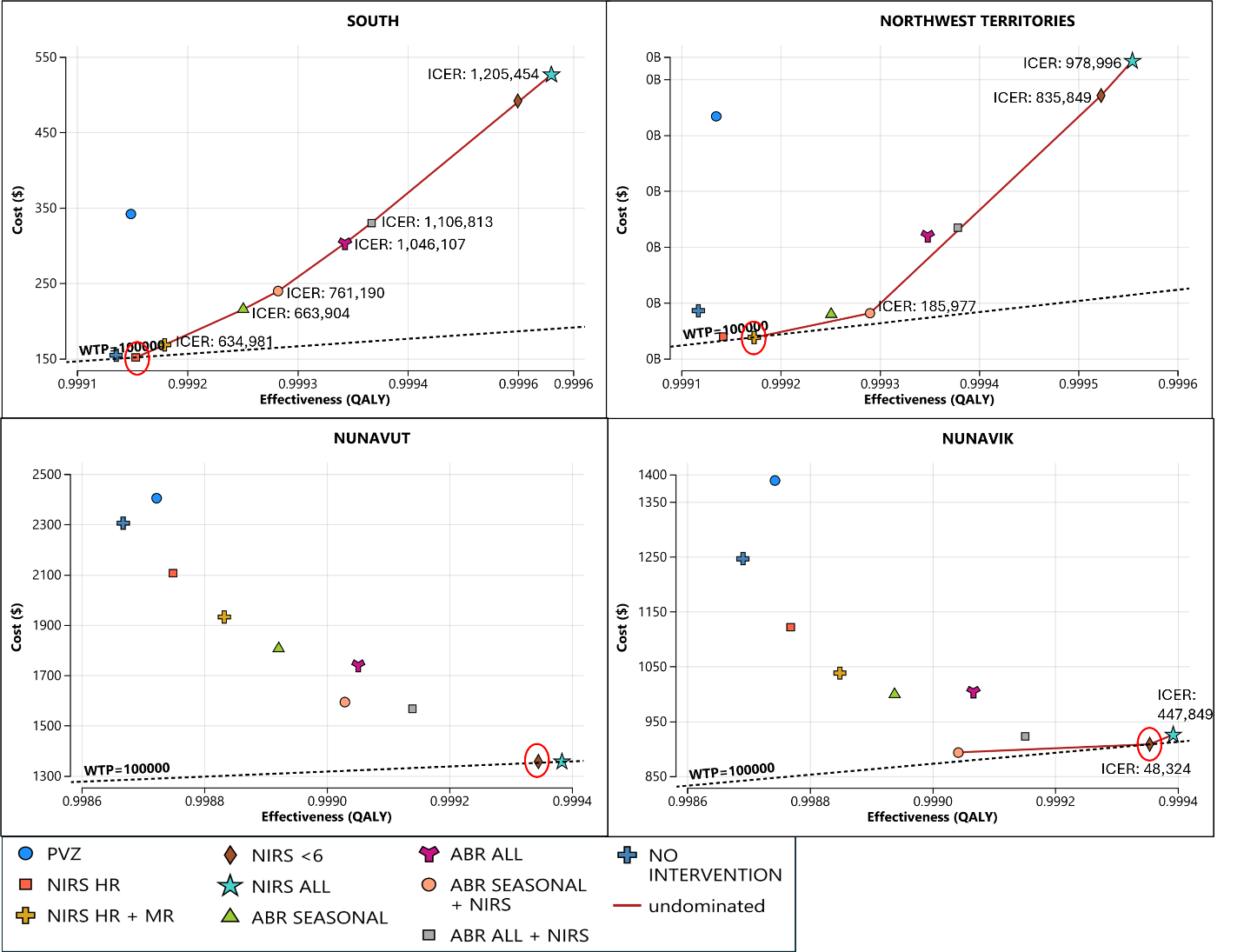


ABR: RSVpreF, HR: high risk, ICER: incremental cost-effectiveness ratio, MR: medium risk, NIRS: nirsevimab, PVZ: palivizumab, QALY: quality-adjusted life year. Costs and QALYs are per infant. ICERs are measured in $/QALY. Red circles indicate most cost-effective strategy.

Figure S3. Cost-effectiveness results from societal perspective


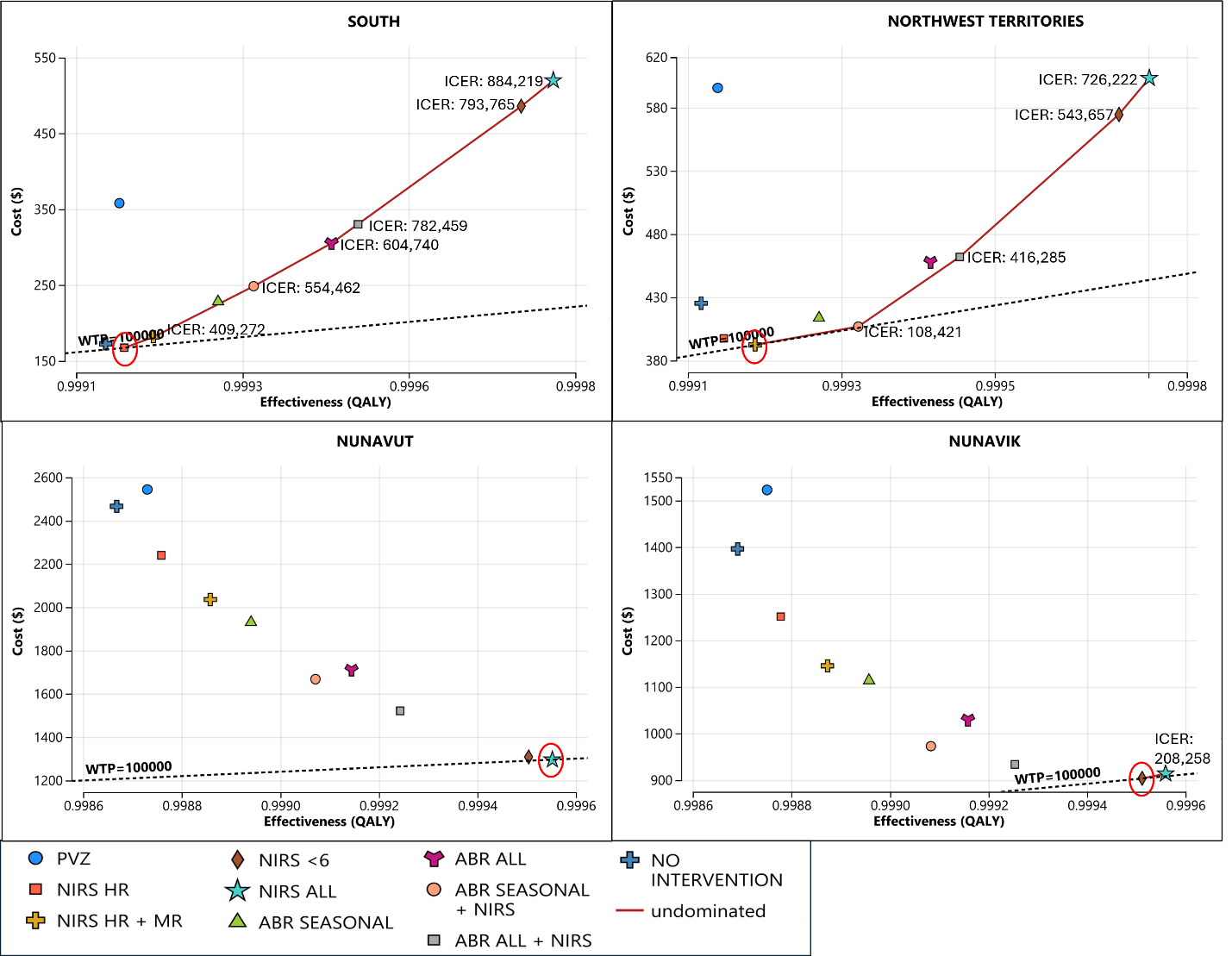


ABR: RSVpreF, HR: high risk, ICER: incremental cost-effectiveness ratio, MR: medium risk, NIRS: nirsevimab, PVZ: palivizumab, QALY: quality-adjusted life year. Costs and QALYs are per infant. ICERs are measured in $/QALY. Red circles indicate most cost-effective strategy.

Figure S4. Cost-effectiveness results from expanded societal perspective*.


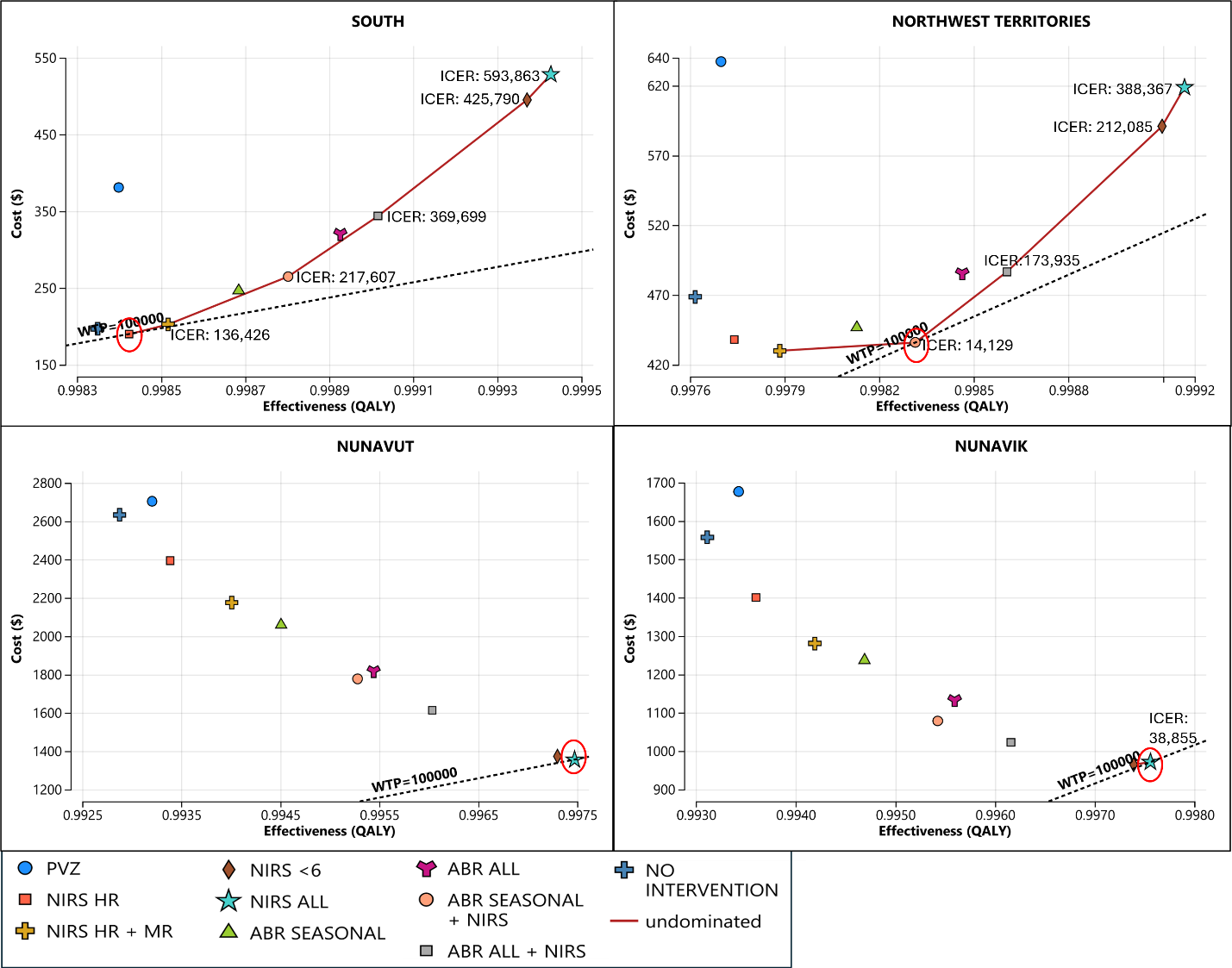


ABR: RSVpreF, HR: high risk, ICER: incremental cost-effectiveness ratio, MR: medium risk, NIRS: nirsevimab, PVZ: palivizumab, QALY: quality-adjusted life year. Costs and QALYs are per infant. ICERs are measured in $/QALY. Red circles indicate most cost-effective strategy.
*Includes monetary loss of life and QALY loss associated with infant mortality.
